# Covalent Conjugates of Allylbenzenes and Terpenoids as Antibiotics Enhancers with the Function of Prolonged Action

**DOI:** 10.3390/ph16081102

**Published:** 2023-08-04

**Authors:** Igor D. Zlotnikov, Maria P. Davydova, Milan R. Danilov, Sergey S. Krylov, Natalya G. Belogurova, Elena V. Kudryashova

**Affiliations:** 1 Faculty of Chemistry, Lomonosov Moscow State University, Leninskie Gory 1/3, 119991 Moscow, Russia; 2 Faculty of Medicine, Lomonosov Moscow State University, Lomonosovsky Prospect 27/1, 119192 Moscow, Russia; 3 N. D. Zelinsky Institute of Organic Chemistry Russian Academy of Sciences, 47 Leninsky Prospect, 119991 Moscow, Russia

**Keywords:** covalent antibacterial conjugate, moxifloxacin, limonene, prolonged pharmacokinetic, FTIR spectroscopy

## Abstract

The drug resistance of pathogenic bacteria is often due efflux pumps—specific proteins that remove foreign compounds from bacterial cells. To overcome drug resistance, adjuvants are often used that can inhibit efflux pumps or other systems that ensure the resistance of bacteria to the action of antibiotics. We assumed that a new level of effectiveness with the use of an antibiotic + an adjuvant pair could be achieved by their joint delivery into the pathogen. To test this hypothesis, we constructed a series of molecular carriers based on poly-(olygo-, dendry)mers based on cyclodextrin-grafted PEI or mannan, as well as glycol chitosan, covalently bound to antibiotic, adjuvant, and the oligosaccharide ligand to the macrophage mannose receptor (CD206), which we studied earlier and showed high efficiency and selectivity of delivery of a therapeutic “cargo” to macrophages. Moxifloxacin was used as an antibiotic, and terpenoid and allylbenzene compounds were used as adjuvants, for which we previously discovered the ability to inhibit bacterial efflux pumps. We show that: (a) the resulting structures were stable in vitro for a long time (up to 10 days); (b) they were adsorbed on bacterial cells, providing a local increase in the concentration of the antibiotic and adjuvant in pathogen cells; (c) they were internalized by bacterial cells, ensuring the accumulation of both antibiotic and adjuvant inside bacterial cells; (d) the adjuvant, after entering the bacterial cell, provided inhibition of the efflux pumps; (e) due to this action of the adjuvant, combined with the targeted delivery by the carrier, the antibiotic’s half-life in rats increased by more than 2 times, the effective concentration of the drug in the blood plasma (AUC) increased up to 8–10 times; (f) a significant increase in the effectiveness of the antibacterial action against Gram+ and Gram- cells was achieved (up to 3 times). Potentially, such an approach would significantly increase the effectiveness of therapies for a number of infectious and other diseases, reduce the dosage of antibiotics, shorten the duration of treatment, and reduce the risk of developing bacterial resistance. Moreover, the use of a polymer carrier with covalently bound organic molecules of different structures will avoid problems linked to different (suboptimal) solubility and bio-distribution of the administered molecules, which would be almost inevitable when using the same compounds separately. It would be very difficult to find antibiotic/adjuvant pairs that simultaneously achieve optimal concentrations in the same target cells. In our case, terpenoids and alkylbenzenes used as adjuvants are practically insoluble as individual compounds, and their unacceptable pharmacological properties would not allow them to be used as efflux pump inhibitors.

## 1. Introduction

Infections caused by pathogenic bacteria are one of the main causes of death in developing countries and a serious health problem in developed countries. The widespread use of antibiotics leads to the emergence and development of resistant and multi-resistant strains, resistant to several types of antibiotics at the same time, as well as to reservoirs of latent or “dormant” bacteria. Multidrug resistance (MDR) is a growing global problem: every year, about 2.5 million people worldwide die from antibiotic-resistant bacterial infections. By 2050, this figure may grow to 10 million people a year. Among the main mechanisms of the emergence of resistance are the poor penetration of antibiotics into the localities of latent infection, as well as the work of efflux pumps—specific proteins that remove drug compounds from bacterial cells [1].

The targeted delivery to macrophages opens up numerous opportunities to influence a wide range of diseases and pathological conditions, of which they are the driver or participant [2,3,4,5,6,7,8,9,10,11,12,13,14,15,16,17,18,19,20,21,22,23,24,25,26,27]. The developed highly effective systems for delivering antibacterial drugs to macrophages are able to increase the effectiveness of therapy by directly affecting the reservoir of latent infection localized in them. Systematic studies of CD206 ligands conducted by our group [6,19,28,29,30,31,32] allowed us to develop a specific molecular container carrying an oligomannoside ligand of complex structure with optimal affinity to the mannose receptors of macrophages. We modeled the interaction of CD206 with more than a hundred relevant carbohydrate structures, of which about two dozen were studied experimentally. As a result, optimized polymer ligands provided the effect of accumulation of a therapeutic “cargo” in macrophages, which significantly increased organ bioavailability (bio-distribution and accumulation of drugs in the lungs) and the permeability of bacterial cells to drugs.

Additionally, a significant increase in efficiency can be achieved by using adjuvants that increase the permeability of bacterial cells to the antibiotic and inhibit the efflux proteins of bacteria (which “throw” the drug out of the cells), which can significantly increase the accumulation of the main drug in bacterial cells. As adjuvants, in our scientific group, compounds of the terpenoid and flavonoid series are extensively studied, which show synergism with the main drug—antibiotics MF, levofloxacin, rifampicin, etc. Terpenoids and flavonoids are extracted from essential oils of plants that exhibit a number of remarkable biological effects [6,28,33,34,35,36,37,38,39,40,41], including analgesic, antibacterial, anti–inflammatory, and antioxidant activity.

However, they are insoluble in aqueous media, which limits their positive effects for practical application. The molecular containers we are developing can allow us to realize their potential and achieve their synergistic effect with antibiotics: when the combined preparation of fluoroquinolone and its adjuvant is included in the delivery system, a dual mechanism of action of the adjuvants is shown: increasing the permeability of bacterial cells to the antibiotic and inhibiting the efflux of bacterial proteins, which allows for an increase in the accumulation of the drugs in bacterial cells. So, for the combination of fluoroquinolone–terpenoid, we observed a 2–3-fold increase in the effectiveness of the antibiotic (a 2–3-fold decrease in MIC) [42,43,44].

However, the binding constants of MF and adjuvants with the developed molecular containers—polymer ligands—are not high enough (about 10^4^ M), which will cause the premature dissociation of the complexes upon intravenous administration and will not provide for a prolonged action of the antibiotic.

In the presented article, we aimed to create a moxifloxacin prodrug—a covalent conjugate of the antibiotic with mannosylated polymers (dendrimers) based on cyclodextrin-grafted PEI or mannan enhanced by a terpenoid adjuvant with the function of prolonging the drug action, promoting a long-term circulation in the bloodstream. As a comparison system, the same antibacterial agents included in polymeric nanogels crosslinked using biodegradable bifunctional agents, such as genipin [45,46,47,48,49,50] or acetylcysteine derivatives, providing the formation of disulfide bonds between the polymer chains, were considered. Potentially, the approach suggested would significantly increase the effectiveness of therapy for a number of infectious and other diseases, reduce the dosage of antibiotics, shorten the duration of treatment, and reduce the risk of developing resistance. Moreover, the use of a polymer carrier with covalently bound organic molecules of different structures will avoid problems due to the different (suboptimal) solubility and bio-distribution of the molecules, which would be almost inevitable when using the same compounds separately. The creation of such formulation is expected to be helpful in the future, in terms of increasing the effectiveness and safety of therapies for infectious diseases such as tuberculosis, pneumonia, pulmonary fibrosis, gastrointestinal inflammation.

## 2. Results and Discussion

### 2.1. Synthesis and Characterization of Covalent and Non-Covalent Antibacterial Formulations

We studied the function of prodrug creations obtained by the covalent attachment of antibacterial agents (moxifloxacin (MF) enhanced by terpenoids or allylbenzene adjuvants) to mannosylated polymers based on cyclodextrin-grafted PEI or mannan as well as glycol-chitosan. The mannose label is important for the targeted delivery of a polymer with an antibacterial drug to macrophages in the sites of localization of pathogens. We expected that covalent conjugates could show prolonged antibacterial action as well as enhanced antibacterial activity due to their higher local concentration in the vicinity of the areas of inflammation; additionally, they could circulate in the blood flow much longer compared to non-covalent antibacterial formulations. An important task was to compare prodrugs consisting of MF conjugated through an amide bond or through an ethereal bond: firstly, from the point of view of optimal synthesis tactics, secondly, by examining the antibacterial activity of these prodrugs (conjugates), and thirdly, by evaluating the duration of the drug’s action as well as the synergistic effect of antibiotics with the adjuvants.

As an alternative option for creating a composition (MF + adjuvant) with the prolonged action and increased efficiency, we propose an approach based on the inclusion of the drug in a gel, followed by crosslinking with biodegradable and stimulus-sensitive bifunctional crosslinking agents (spontaneously and reversibly forming disulfide bonds or forming imide bonds between amino groups of polymers (PEI, chitosan) and genipin). In this case, the MF formulation was realized without the involvement of functional groups of antibiotics in the covalent coupling.

Table 1 shows the names and chemical designations of MF conjugates, with adjuvants and polymers.

To realize this strategy, we focused on the following points:

(1) Synthesis and characterization by FTIR and NMR spectroscopy of the covalent conjugates of MF, allylbenzenes (EG and apiol), terpenoids (linalool and limonene) with polymers (based on PEI, chitosan, mannan), with a variable type of covalent linkage (amide or ester bond) and with a variable crosslinking agent, to determine the most efficient and optimal composition of the antibacterial conjugate.
(2) Incorporation of the drug composition into polymer nanogels followed by covalent crosslinking of the particles, without involving the drug in covalent bonds.
(3) Investigation of the antibacterial activity (against *E. coli* and *B. subtilis*) of the developed prodrugs in terms of efficiency and duration of their action.
(4) Elucidation of the mechanisms of the conjugate action in cells using FTIR spectroscopy in comparison with free antibacterials.
(5) Study of the pharmacokinetics of the antibacterial covalent prodrugs in comparison with the antibacterial formulations included in nanogels (non-covalent prodrugs).

#### 2.1.1. MF Covalent Prodrug Formulations Synthesis and Characterization by FTIR and NMR Spectroscopy

**Figure 1:**
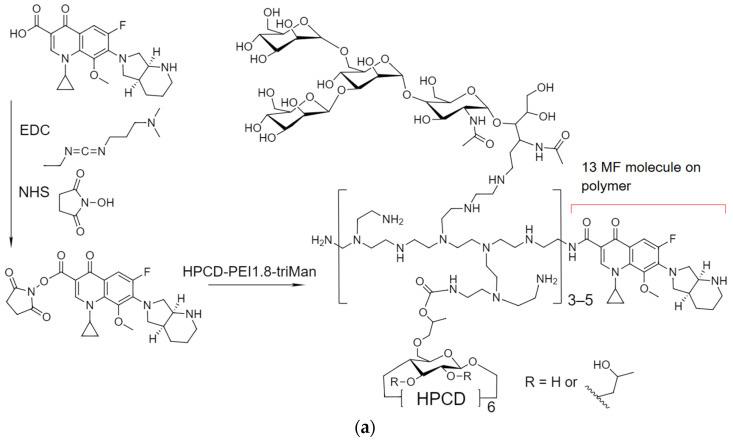

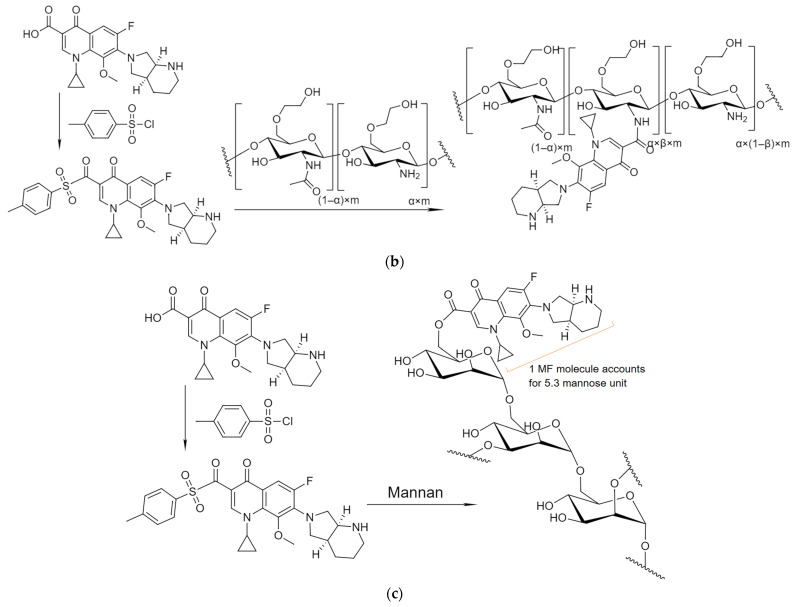

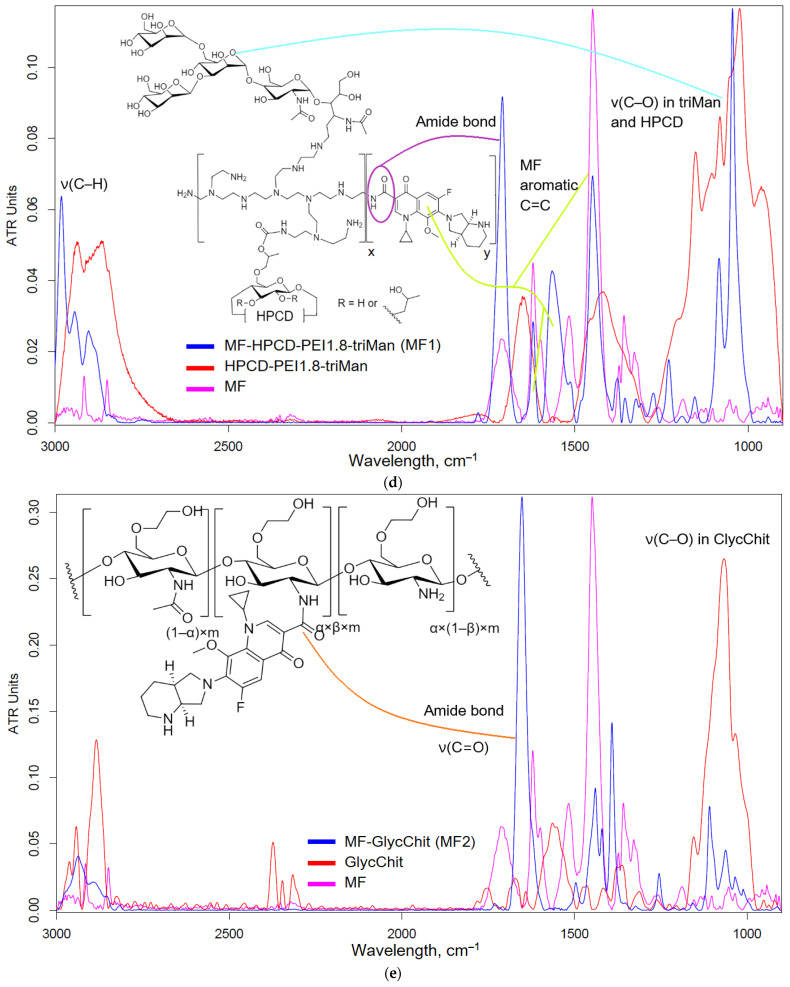

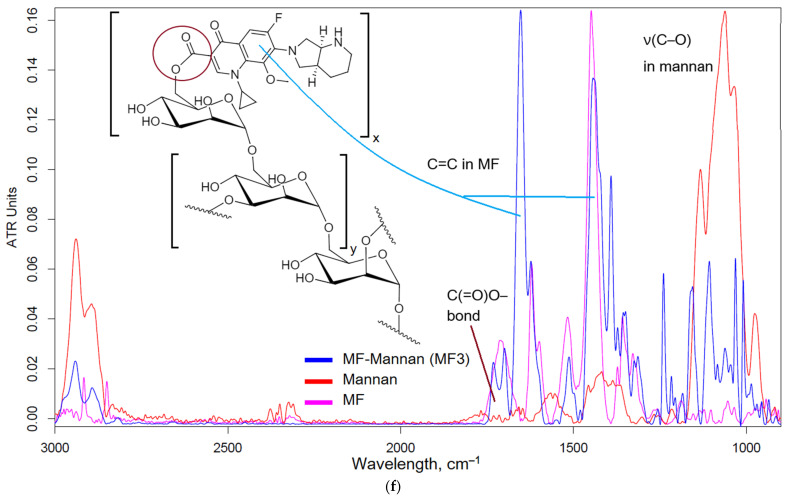

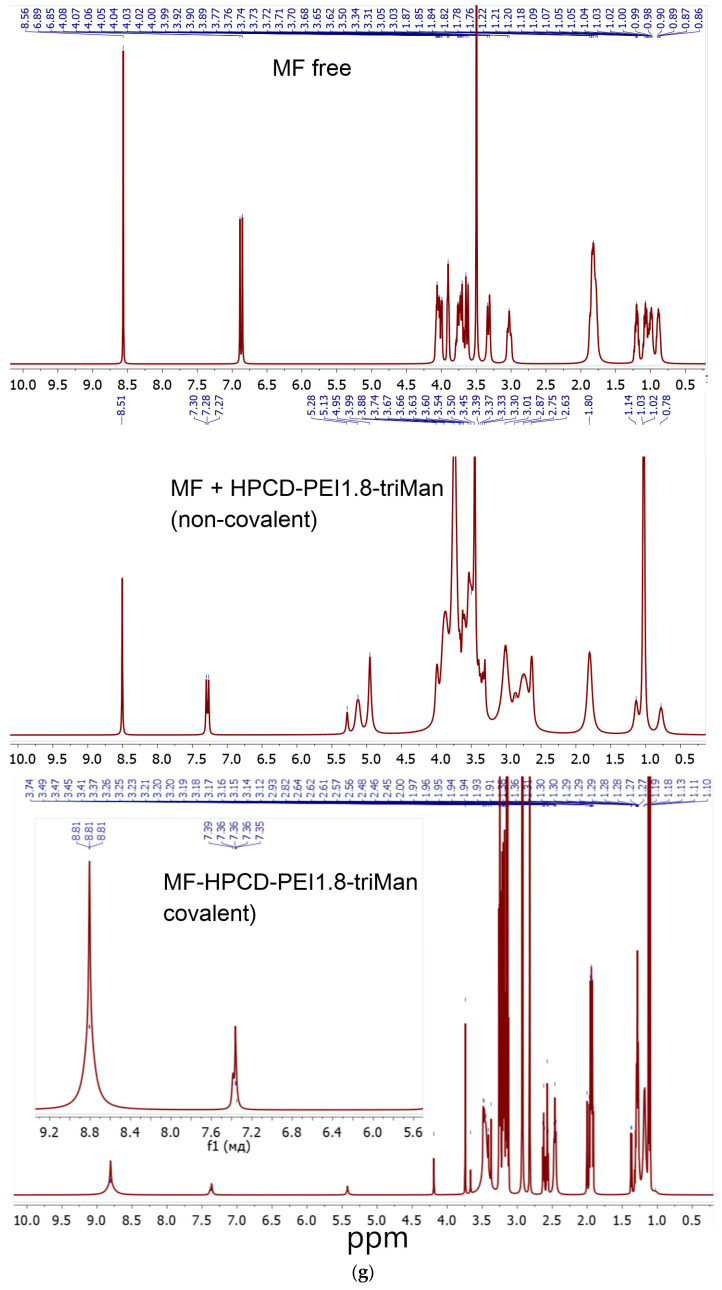

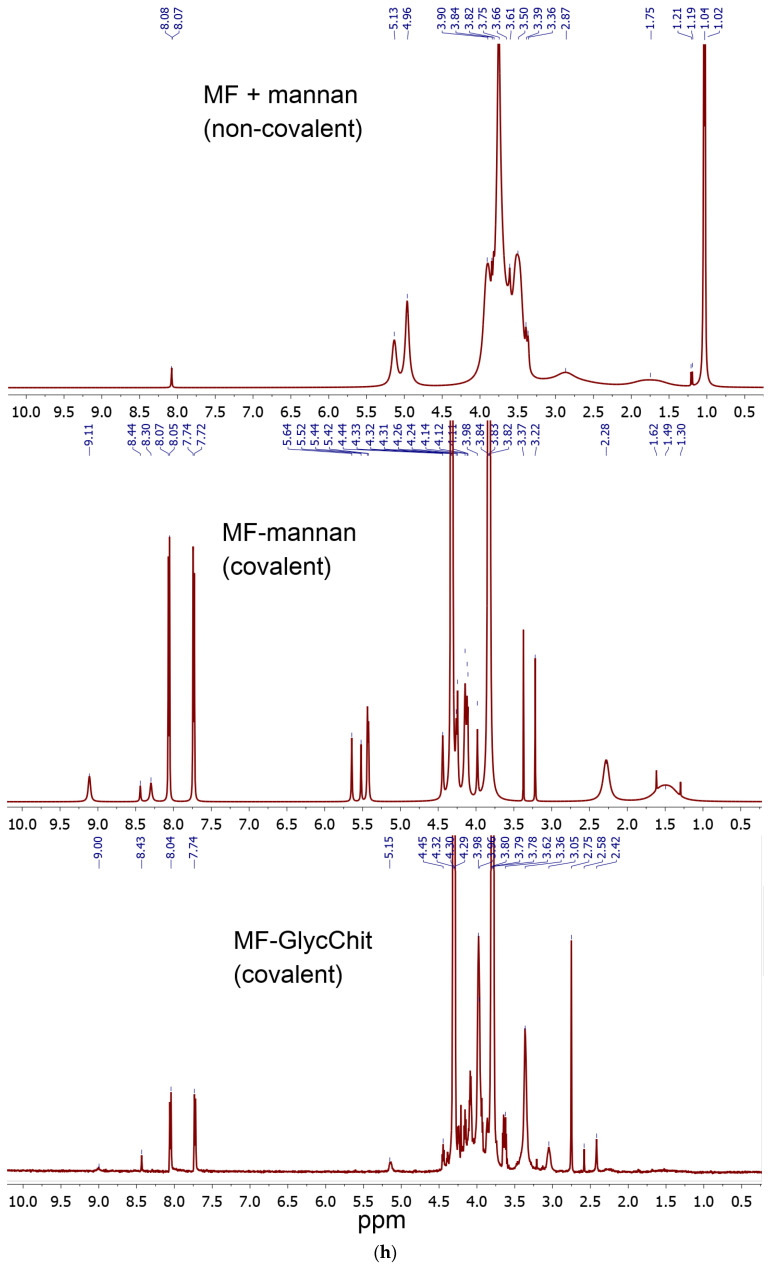

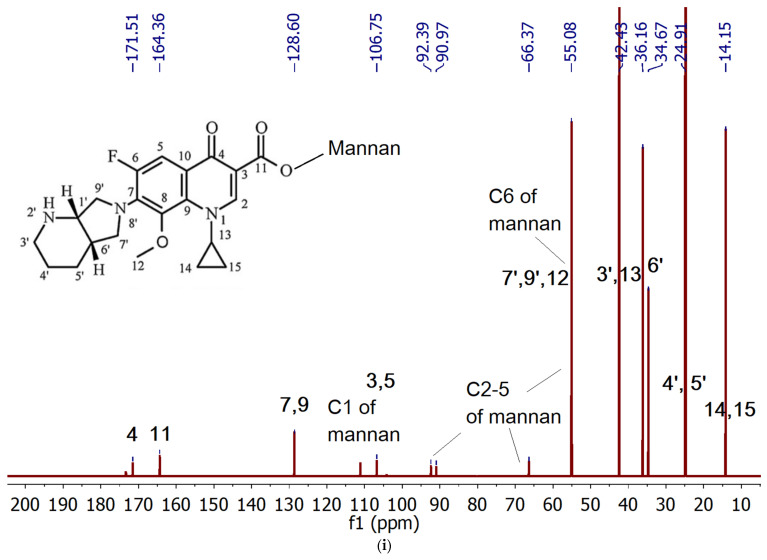
The scheme of the synthesis of MF–polymer conjugates based on: (**a**) HPCD–PEI1.8-triMan, (**b**) GlycChit, (**c**) Mannan. FTIR spectra of MF, polymers and MF–polymer conjugates based on (**d**) HPCD–PEI1.8-triMan, (**e**) GlycChit, (**f**) Mannan. PBS (0.01 M, pH 7.4). T = 22 °C. ^1^H NMR spectra of MF, mixtures and MF–polymer conjugates based on (**g**) HPCD–PEI1.8-triMan, (**h**) GlycChit and Mannan. D_2_O. (**i**) ^13^C NMR spectra of MF–mannan (MF3). T = 25 °C.

Figure 1a–c shows a scheme for the synthesis of MF conjugates with polymers. Figure 1d–f shows the FTIR spectra and structures of the conjugates MF–polymer. The crosslinking of MF and polymers was realized through the formation of amide (in the case of HPCD–PEI1.8-triMan and GlycChit) or ester (in the case of mannan) bonds (Figure 1a–c), which have characteristic peaks in the FTIR spectra, at 1710, 1650 and 1700–1740 cm^−1^, corresponding to valence oscillations of C=O (in the specified order, amide bond, corresponding to the peak at 1650 cm^−1^ (Figure 1d,e) and ester bond visible by splitting the peak at 1710–1740 cm^−1^ (Figure 1f)). The FTIR spectra of the conjugate contained peaks characteristic of both MF (ν(C=C) 1400–1600 cm^−1^, ν(C–H) 2850–2970 cm^−1^) and the polymers (C-O-C 1000–1100 cm^−1^). It is assumed that the ester bond is easier to hydrolyze than the amide bond, which is more stable and is hydrolyzed enzymatically by plasma proteases, potentially affecting the properties of the conjugates in terms of antibacterial activity and pharmacokinetic parameters.

The successful synthesis was confirmed by NMR spectroscopy data (Figure 1g,h): both polymer signals (2.5–4.0 and 5.0–5.5 ppm) and characteristic signals for aromatic MF protons (7.2–9.0 ppm) were present in the composition. With the covalent crosslinking of MF, the MF signals shifted into a weak field. Figure 1i shows the ^13^C NMR spectrum of the MF–mannan conjugate with the indicated assignment of the peaks [51,52], characteristic of MF, mannan and the ester bond between the two components. The ^1^H and ^13^C NMR spectra of intermediate compounds are presented in the appendix (Figure S1).

#### 2.1.2. Allylbenzenes and Terpenoids Formulations

Some components of plant oils (eugenol (EG) and terpenoids) demonstrate antibacterial activity, which is weaker in comparison with that of classical antibiotics, but sufficient to partially replace them, i.e., reduce the dosage of toxic drugs and have an enhancing effect on the main component (in this work, MF). One of the tasks of this work was to obtain soluble forms of allylbenzenes and terpenoids due to their conjugation with polymers or inclusion in polymer particles.

**Figure 2:**
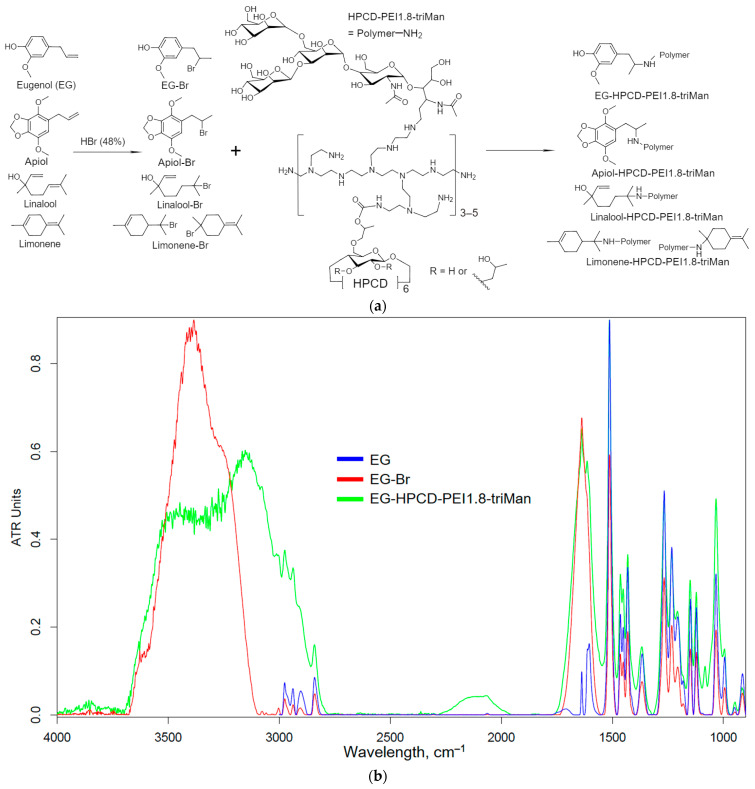

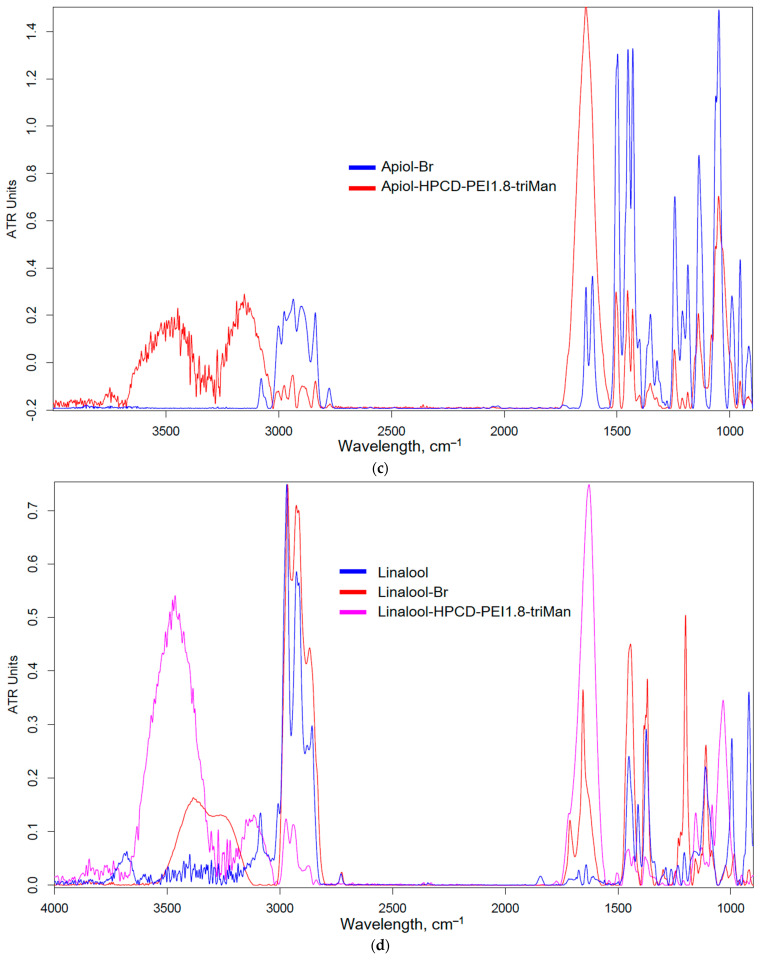

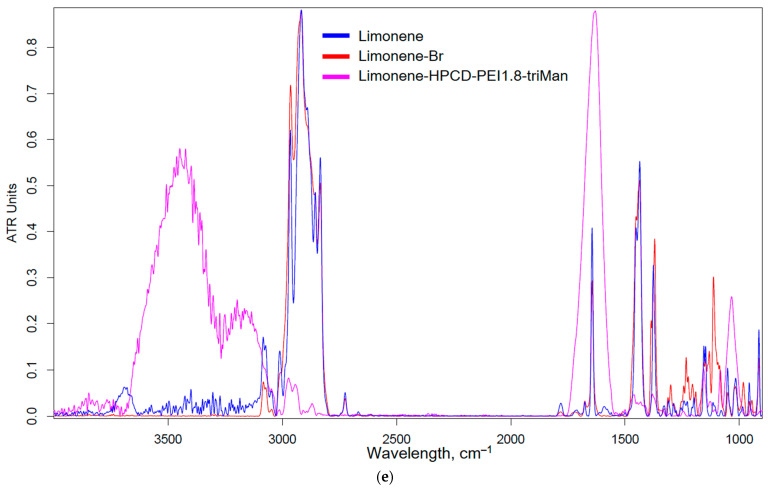

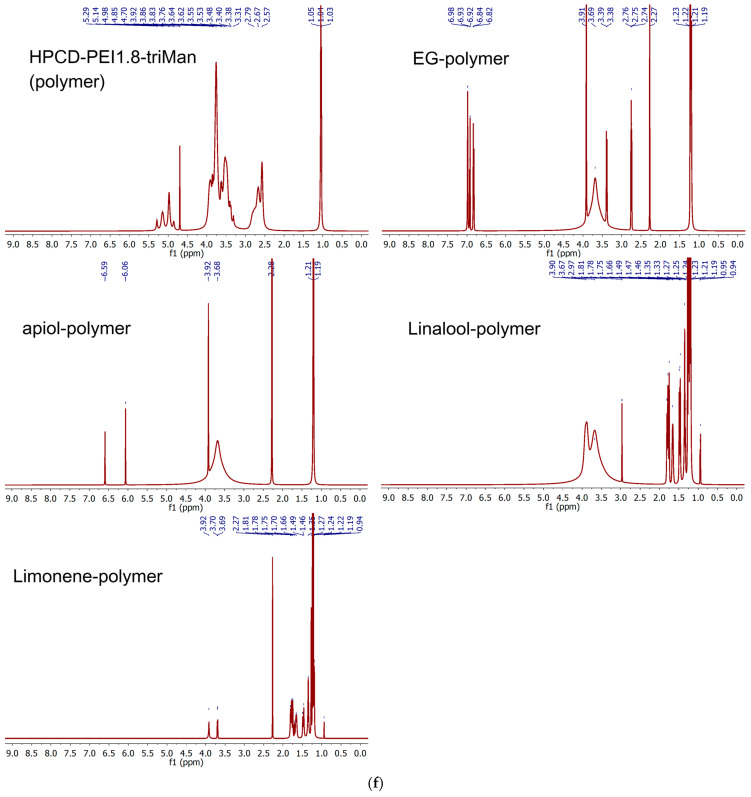
(**a**) Synthesis scheme and FTIR spectra of (**b**) EG, (**c**) apiol, (**d**) linalool, (**e**) limonene, their bromine derivatives and conjugates with polymers. T = 22 °C. (**f**) ^1^H NMR spectra of HPCD–PEI1.8–triMan and HPCD–PEI1.8–triMan grafted by apiol, EG, linalool, limonene. D_2_O. T = 25 °C.

Figure 2a shows a scheme for the synthesis of covalent conjugates of EG, apiol, linalool and limonene with HPCD–PEI1.8–triMan. The first stage was the addition of HBr to C=C, accompanied by a change in shape (broadening) and an increase in the intensity of FTIR peak at 1600–1800 cm^−1^ (Figure 2b–e), characteristic of ν(C=C) oscillations in an aromatic system or double bond. The second stage was the nucleophilic substitution of a bromine atom in the allylbenzene or terpenoid molecule by an amino-group of the HPCD–PEI1.8–triMan polymer, accompanied by the appearance or change of a peak at 3000–3600 cm^−1^ (valence oscillations of O–H, N–H) due to the contribution of the polymer functional group. An increase in the intensity of the peak corresponding to C-O-C at 1000–1100 cm^−1^ also indicated the presence of a polymer in the final product.

Figure 2f shows the ^1^H NMR spectra of the HPCD–PEI1.8–triMan polymer, as well as of its grafted derivatives with apiol, EG, linalool, limonene. Characteristic peaks were observed in the polymer spectrum for the HPCD protons H1 (5.0–5.4 ppm), H2-H6 (3.3–4.0 ppm), the PEI protons –NH–CH_2_–CH_2_– (2.5–3.0 ppm), the CH_3_-group (1.0–1.1 ppm). In the NMR spectra of the modified polymer by apiol and its analogues, peaks characteristic of the aromatic protons of apiol and EG were observed (6.0, 6.6 ppm for apiol, 6.8–7.0 ppm for EG) and there was no peak at 5–6 ppm corresponding to the C=C double-bond protons, which indicated a successful modification. In the spectra of linalool and limonene attached to the polymer, patterns characteristic of the polymer itself were observed, as well as patterns characteristic of the hydrocarbon skeleton of these adjuvants.

Thus, FTIR and NMR spectroscopy confirmed the formation of covalent conjugates of allylbenzenes, terpenoids and MF with polymers. However, a covalent attachment can lead to a loss or decrease in biological activity; so, for comparison (as a control system,) we also synthesized covalently crosslinked nanogels where the antibiotic and its adjuvants were non-covalently included in the formulations, as discussed above.

#### 2.1.3. Non-Covalent Formulations of MF, Allylbenzenes and Terpenoids with Glycol–Chitosan Hydrogels Stabilized by Crosslinking Bifunctional Agents

**Figure 3:**
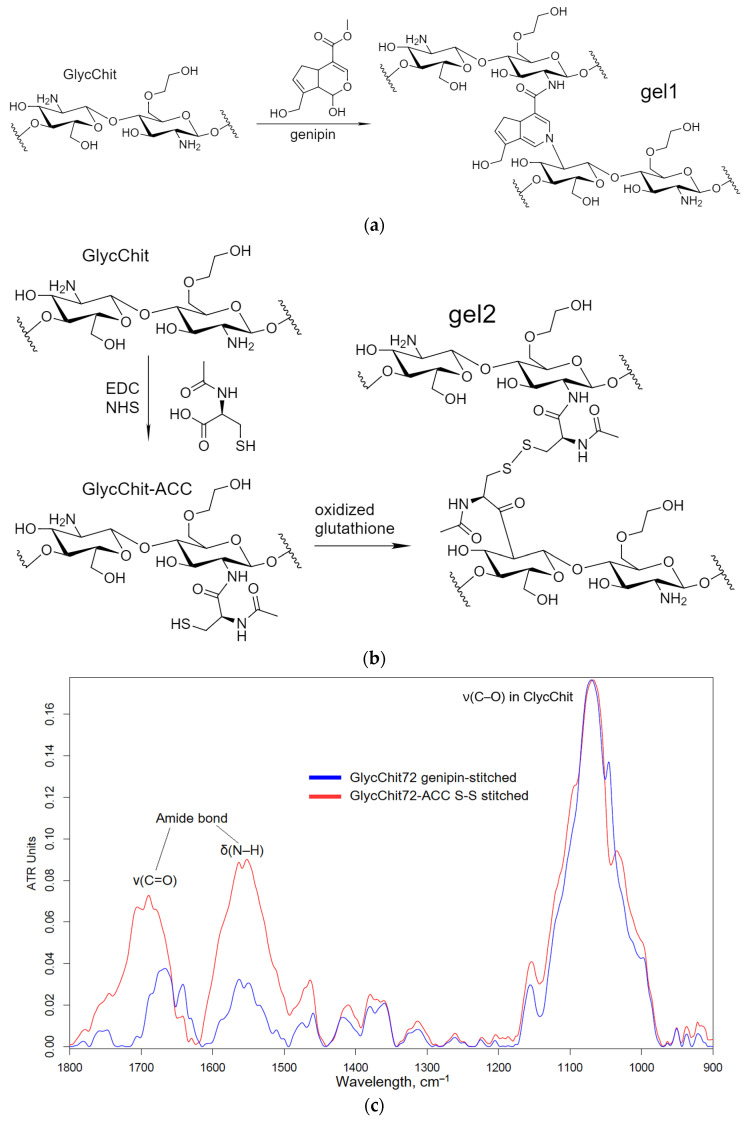
The scheme of the synthesis of (**a**) genipin-stitched GlycChit, (**b**) N-acetylcysteine-stitched GlycChit and their FTIR spectra (**c**).

Non-covalent formulations of combined antibacterials were obtained by including antibacterial agents into polymer particles, followed by crosslinking the polymer chains with each other. For such systems, it is expected that the biological (anti-inflammatory, antioxidant, antibacterial, antitumor) properties are not lost or, even, they are increased. A number of crosslinking agents have been described in the literature: glutaraldehyde [53,54], diisocyanates [55]; however, they have toxic properties. In this work, biocompatible and even medically “useful” crosslinking agents were used, i.e., genipin [56,57,58,59,60,61,62,63] and N-acetylcysteine. Genipin (from clove oil) is able to crosslink chitosan amino groups in different chitosan polymer chains due to the formation of amide and amine bonds (Figure 3). As a second variant, we propose a new pH- and stimulus-sensitive acetylcysteine-cross-linked (attached to chitosan by the carbodiimide method) chitosan with the formation of S-S bonds (a nanogel forms spontaneously when chitosan polymeric chains fold, and this reaction is reversible and can be regulated by changing the conditions).

### 2.2. Antibacterial Activity of MF, Allylbenzenes, Terpenoids

#### 2.2.1. Primary High-Throughput Screening of the Antibacterial Activity of Prodrug Conjugates

To find the most efficient antibacterial agent or combination, we conducted a primary screening of the activity of MF as well as of allylbenzenes and terpenoids conjugates in covalent prodrugs and in non-covalent nanogels, focusing on the samples that demonstrated pronounced antibacterial activity.

**Figure 4:**
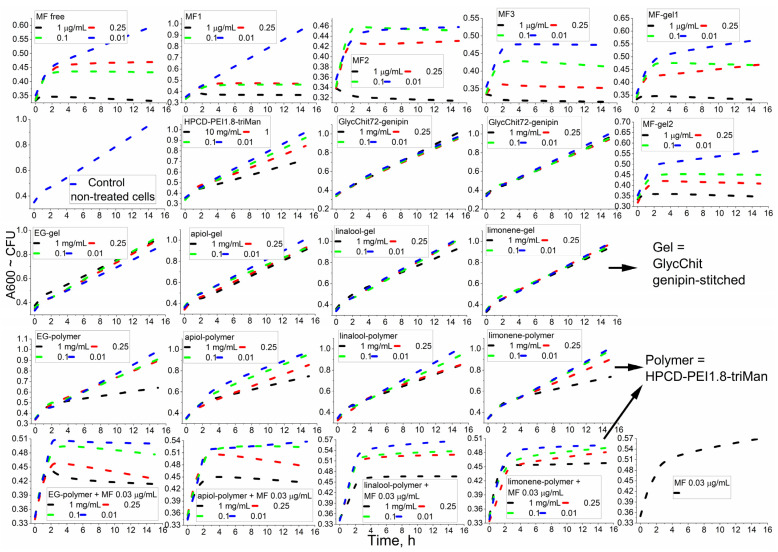
Kinetic curves of the dependence of the optical density at 600 nm, correlating with the number of colony-forming units, on the incubation time (0–16 h) of *E. coli* cells with antibacterial drugs, at 37 °C. Line 3: the gel was GlycChit genipin-stitched. Lines 4, 5: polymer = HPCD–PEI1.8–triMan. MF-gel1 = MF + GlycChit genipin-stitched. MF-gel2 = MF + GlycChit–acetylcysteine S-S-stitched. The chemical designations of MF conjugates, adjuvants and polymers are indicated in Table 1.

Figure 4 shows the curves for antibacterial formulations of cell growth dependence on the incubation time with—as a primary screening analysis of the effectiveness of MF and adjuvants. Table 2 and Table 3 show quantitative characteristics of the antibacterial activity of the covalent prodrugs (in conjugates) and of the prodrug in nanogels. In the case of MF, as a result of the covalent attachment to the polymer, the value of half maximal inhibitory concentration (IC50) against *E. coli* decreased due to the increased adsorption of MF, which resulted in higher drug efficacy [64]. This effect was clearly observed against Gram-positive *B. subtilis*, against which free MF (non-formulated) was much less active compared to the MF is conjugate.

The effectiveness of the drug was also characterized by determining the kinetic curves of bacterial growth (Figure 4). Strong suppression of bacterial growth (MF conjugates) and a plateau and/or a decline in CFU for powerful antibacterial agents (MF and its non-covalent gel formulations) were observed. For the samples (low concentrations of MF and high concentrations of the adjuvants components, EG, etc.) we observed that cell growth slowed down. Finally, the effect was hardly noticeable for chitosan itself and the non-covalent formulations of linalool and limonene.

Among the individual components of terpenoids and allylbenzenes, the most effective were EG against *E. coli*, as well as EG, limonene and apiol against *B. subtilis*. In addition, EG and its analogues demonstrated an amplifying effect for MF (Table 3) by 10–15% in terms of CFU (cell viability), suggesting a potential application of this molecule to reduce the dose of toxic antibiotics.

Polymers themselves showed a weak antibacterial effect (Figure 4, line 2), the strongest being that of the PEI-containing polymer. The covalent conjugates of MF with polymers (Figure 4, line 1), as well as of components of essential oils with polymers (Figure 4, line 4), turned out to be more active than non-covalent chitosan-based “gels” (Figure 4, MF-gel1, MF-gel2 and line 3). However, using nanogel formulations, it was possible to achieve a prolonged release of the antibacterial drugs without modification of the drug itself. We previously showed that the formation of chitosan particles is accompanied by the inclusion of hydrophobic substances, the delivery of which to bacteria is improved due to their high concentration and adsorption on the cell membrane. Thus, cross-linked polymers (“chitosan nanogels”) have the potential to deliver antibacterial drugs compositions.

#### 2.2.2. Prolonged Antibacterial Experiment with the Most Promising Formulations

**Figure 5:**
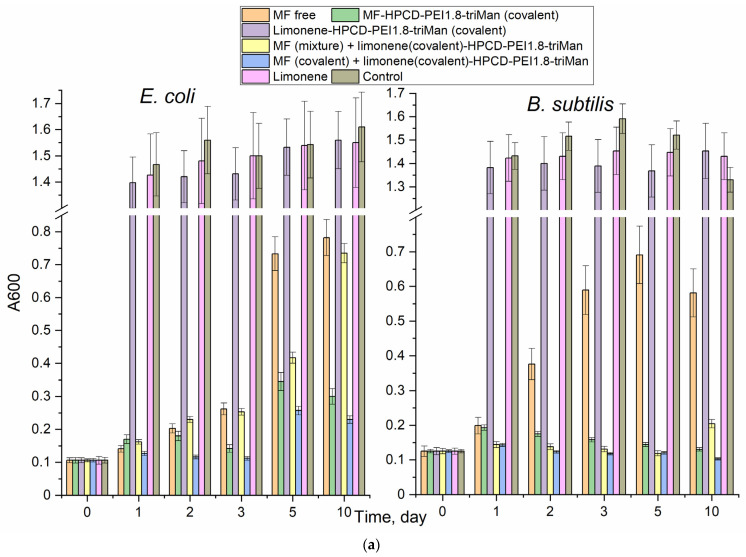

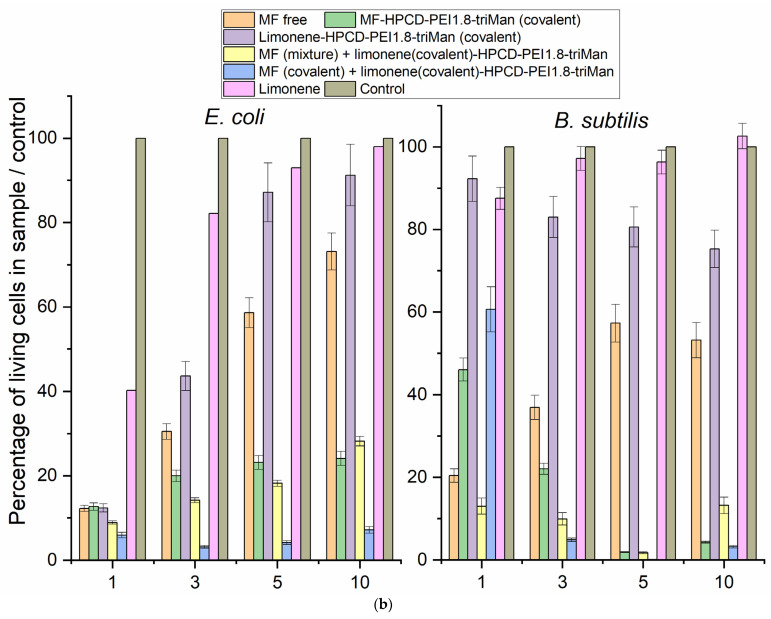
(**a**) The dependence of the optical density at 600 nm, correlating with the number of colony-forming units, on the incubation time for *E. coli* and *B. subtilis* cells treated with antibacterial drugs. (**b**) The ratio of living cells in the sample with respect to the control, determined by DAPI staining. C(MF) = 0.03 μg/mL. C(limonene) = 0.25 mg/mL. 37 °C.

After extensive screening, for a prolonged “antibacterial experiment”, we selected covalent (with higher antibacterial activity) HPCD–PEI1.8–triMan and MF or linalool non-covalent formulations for comparison. Figure 5 shows the dependence of survival (determined by A600 measuring and DAPI staining of dead cells [65], with an additional control consisting of cells seeded on Petri dishes without treatment) of Gram-positive and Gram-negative cells on the incubation time in the presence of antibacterial agents. We observed that MF free and MF non-covalently included in the polymer nanogel inhibited cell growth, but not completely, as the bacteria continued to grow. We specially used such a concentration of MF to differentiate the observed effects. The MF-HPCD-PEI1.8-triMan conjugate was found to be the most effective against both types of bacteria. Limonene in free form had a weak inhibitory effect on cell growth and could be used to enhance ab antibiotic action in the form of a covalent conjugate, as shown by the excellent results obtained in combination with the covalently bound MF-prodrug (Figure 5b).

MF did not act for more than 2–3 days; then, a significant growth of bacteria was observed (Figure 5a). At the same time, MF in the conjugates, as well as in the presence of the adjuvant limonene, suppressed the growth of *E. coli* (up to 5% cell viability) and *B. subtilis* (completely). MF was degraded after 2–3 days of incubation in an environment with cells that secreted enzymes [1]. MF biodegradation is associated with the elimination of its antibacterial properties, may even lead to the development of resistant bacterial strains.

### 2.3. FTIR Spectroscopy as a Tool for Tracking Drug Penetration into Cells and Its Effectiveness

We showed the effectiveness of covalent conjugates in comparison with non-covalent ones; therefore, it was necessary to show which mechanisms (factors) caused an increase in the antibacterial activity. FTIR spectroscopy provides valuable information about molecular details of drug interactions with cells. The main structural units of a cell are characterize by peaks in the FTIR spectrum corresponding to cell membrane lipids (2800–3000 cm^−1^), proteins—especially transmembrane proteins—(1500–1700 cm^−1^), DNA phosphate groups (1240 cm^−1^), carbohydrates, including lipopolysaccharides (900–1100 cm^−1^) [32]. The main oscillations of bonds in the structural units of *E. coli* cells are observed at 2960–2850 cm^−1^ for CH, CH_2_, CH_3_ in fatty acids, 1655–1637 cm^−1^ for amide I bands (α-helical and β-pleated sheet structures), 1548 cm^−1^ for the amide II band, 1515 cm^−1^ for the aromatic band, 1465–1470 for C–H deformation, 1310–1240 cm^−1^ for amide III band components of proteins, 1250–1220 and 1084–1088 cm^−1^ for the P=O stretching of PO_2_^−^ hosphodiesters, 1100–900 cm^−1^ for the C–O–C, C–O oscillations of the saccharide ring [66].

**Figure 6:**
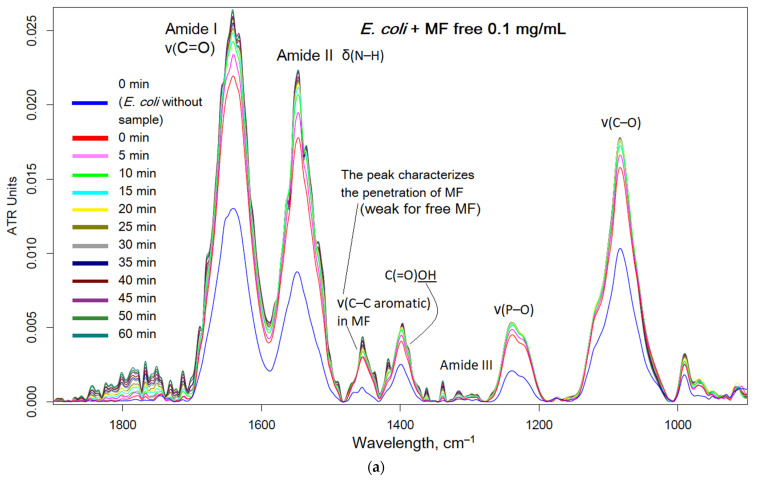

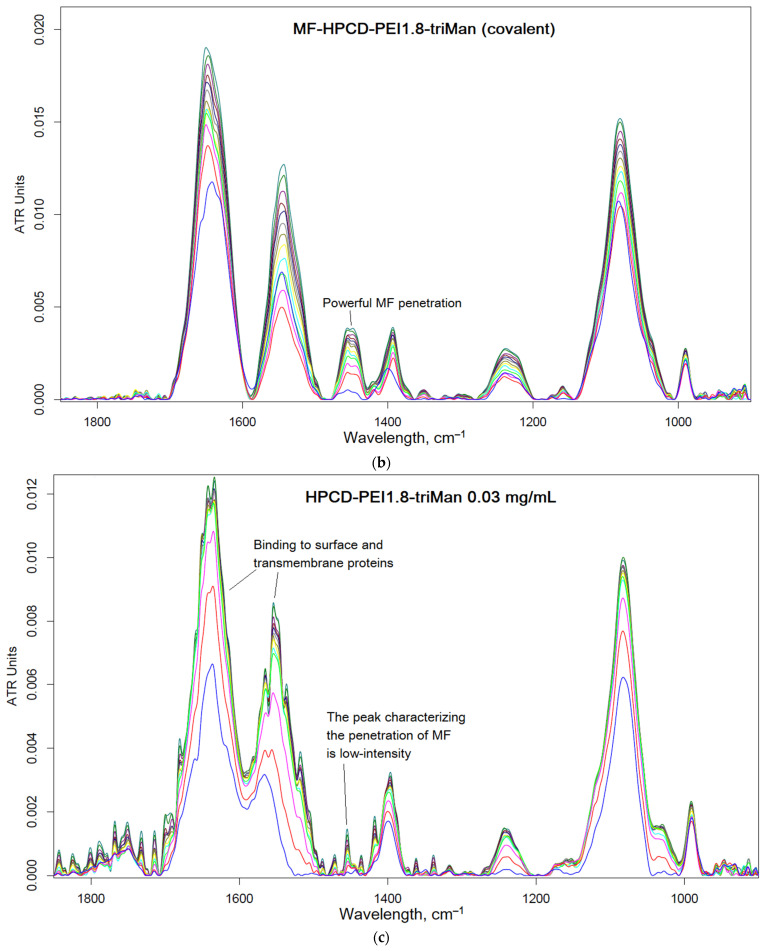

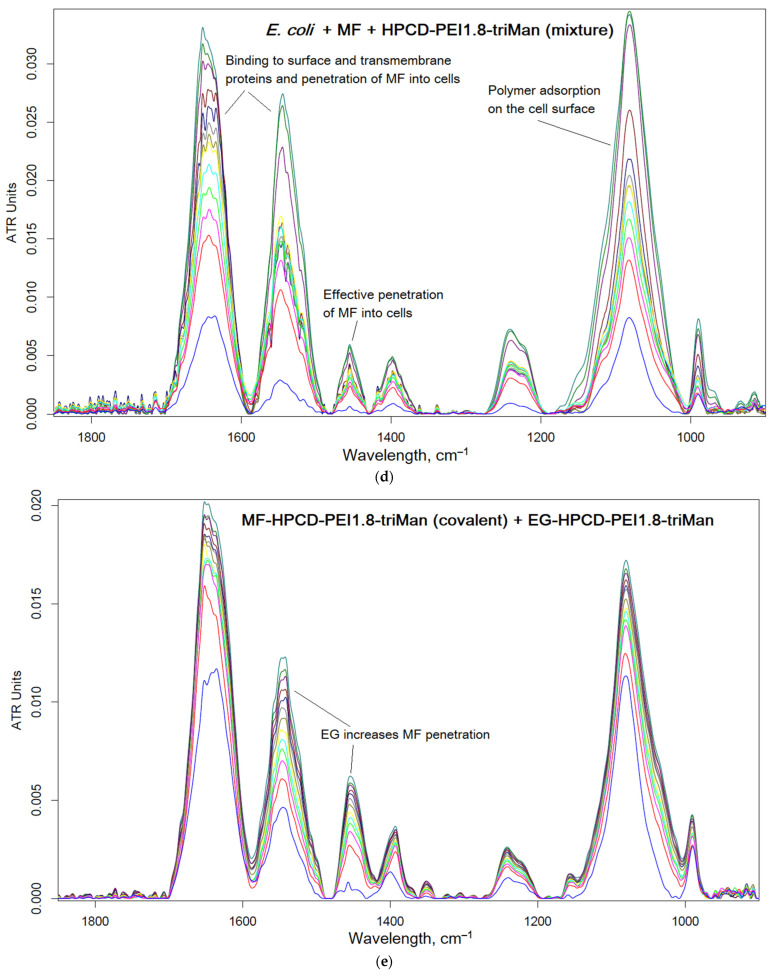

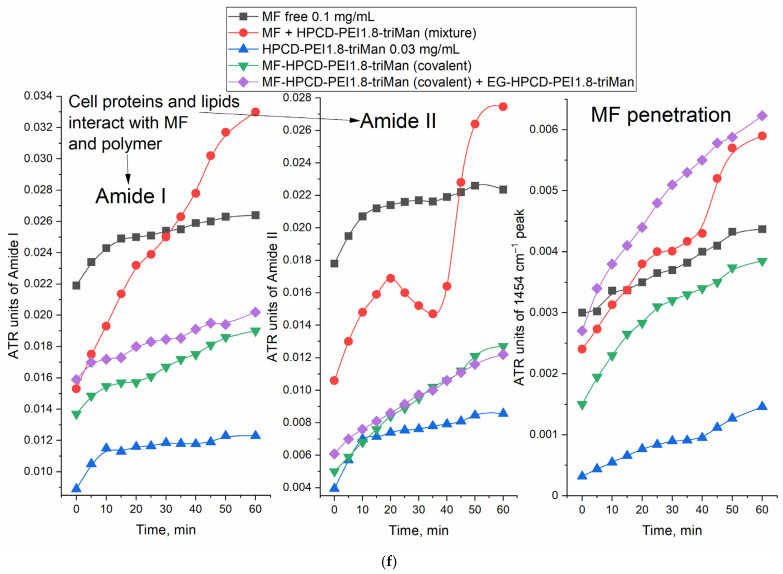
FTIR spectra of *E. coli* cell suspension in PBS during incubation at 37 °C with: (**a**) MF free, (**b**) MF–HPCD–PEI1.8–triMan, (**c**) HPCD–PEI1.8–triMan, (**d**) a mixture of MF with HPCD–PEI1.8–triMan, (**e**) MF–HPCD–PEI1.8–triMan and EG–HPCD–PEI1.8–triMan. C(MF) = 0.1 mg/mL. C(polymer) = 0.03 mg/mL. C(EG) = 1 mg/mL. The color designations are similar for (**a**–**e**) images and correspond to those shown in (**a**). (**f**) The corresponding dependence of the intensity of the FTIR peaks on the incubation time for different samples.

Moxifloxacin is characterized by an intense band at 1454 cm^−1^, corresponding to valence oscillations of the C=C bond of the aromatic system sensitive to the microenvironment. When adsorption on the cell surface or penetration inside the cell occur, an increase in this peak is expected. Figure 6 shows the FTIR spectra of *E. coli* incubated with MF-containing formulations and polymers. The interaction of MF with cell components (proteins, lipids in the membrane, oligosaccharides on the surface) led to an increase in the intensity of the peaks. The most striking changes due to the penetration of MF and polymer adsorption occurred in the bands of amide I and amid II, which characterize proteins. In addition, the peak characteristic of MF is at 1545 cm^−1^, representing, practically, only MF. The graphs (Figure 6f) show the critical dependence of the intensity of the MF peaks in the spectra on the incubation time, as a direct indicator of the effectiveness of drug accumulation in the cell. The changes in the amide I and amide II bands were greatest for unconjugated MF, due to its high molecular mobility. The obtained results are consistent with microbiological data: the covalent conjugate with MF (HPCD–PEI1.8–triMan, with an amide bond) turned out to be the most effective, and an additional increase in the penetration into the cell could be achieved by adding the adjuvant eugenol and other allylbenzenes and terpenoids (a similar effect was observed) as permeability enhancers and efflux inhibitors.

Thus, using FTIR spectroscopy, the high molecular mobility of free MF and its interaction with proteins were shown. At the same time, due to the adsorption of the polymer on the cell membrane and the high local concentration of MF on the polymer, the penetration of MF into the cells increased.

Thus, the most active was the covalent formulation of MF–HPCD–PEI1.8–triMan with the addition of adjuvants (EG and analogue conjugates with polymers); the less active were the non-covalent prodrugs based on MF and chitosan nanogels.

### 2.4. FTIR Spectroscopy as a Tool for Determining the Number of Cells in Antibacterial Experiments

**Figure 7:**
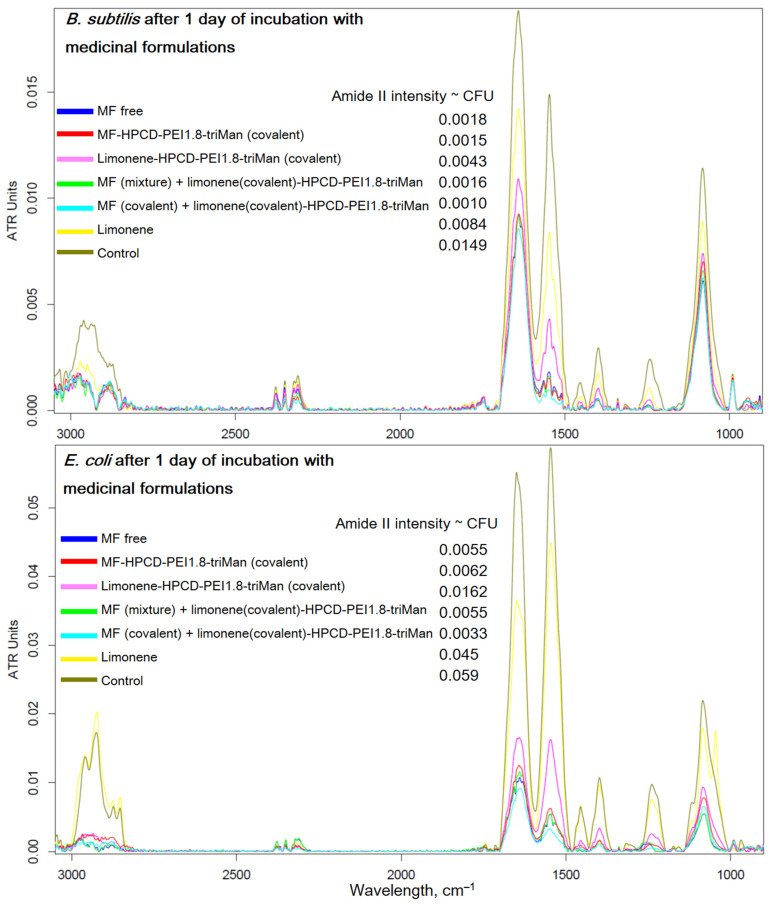
FTIR spectra of *E. coli* and *B. subtilis* cell suspensions in PBS after 1 day of incubation at 37 °C with samples similar to those used in the experiment described in Figure 5.

Above, we showed the applicability of FTIR spectroscopy to monitor the passage of a drug into a cell and discovered the molecular details of the polymer’s action. Here, we aimed to present FTIR spectroscopy from another perspective, i.e., as a means of measuring the number of cells in antibacterial experiments and studying the state of cells by their structural elements. Figure 7 shows the FTIR spectra of bacteria recorded after 1 day of incubation with antibacterial drugs in a prolonged experiment (Section 2.2.2). In the case of *E. coli* incubated with MF–HPCD–PEI1.8–triMan, after centrifugation, we did not observe intact cells (blue curve, PBS spectrum, no intact cells). A small number of cells grew in the presence of MF free and a mixture of MF with polymer. The conjugation of limonene significantly enhanced the antibacterial effect of MF + adjuvant due to the positive charge of the polymer, as well as the high local concentration of the adjuvant. In the case of *B. subtilis*, a similar pattern was observed; however, complete inhibition was not achieved after 1 day, although MF–HPCD–PEI1.8–triMan is also the most active sample. The MF conjugate was enhanced by the presence of limonene in the conjugate, which was reflected in the intensity of the peak of amide 2 (Figure 7). The number of cells (according to the intensity of the amide 2 peak) corresponded to antibacterial data (Figure 5).

Therefore, FTIR is a highly informative method for monitoring the interaction of drugs with individual components of cells (Figure 7). Polymers are adsorbed on the cell surface, increasing the efficiency of MF penetration inside the cells. In addition, FTIR makes it possible to evaluate the effectiveness of antibacterial formulations in real time due to the direct control of the number of cells.

### 2.5. Pharmacokinetics of MF in Polymer Particles and Covalent Conjugates

**Figure 8:**
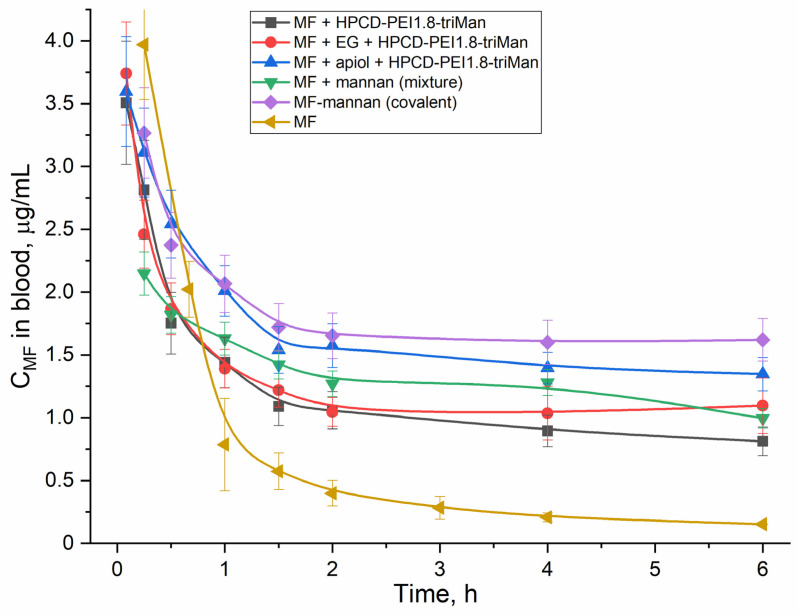
Pharmacokinetic curves of MF and its combinations with polymers (covalent and non-covalent forms) enhanced with adjuvants (EG, apiol) determined for white mongrel rats.

An important feature of essential oil components (EG, apiol, linalool and limonene) is their ability to influence the pharmacokinetics of an antibacterial drug. This is especially noticeable for covalent conjugates. An important aspect of this study was the comparison of non-covalent and covalent MF formulations. Figure 8 shows the pharmacokinetic curves of MF concentration in rat blood plasma. Table 4 shows the corresponding pharmacokinetic parameters using the two-compartment model. In all formulations of MF, the circulation time of MF increased and clearance decreased, while the greatest effect was achieved for the covalent conjugate MF–mannan and for MF included in cyclodextrins as part of HPCD–PEI1.8–triMan, enhanced with apiol, which inhibits a family of enzymes, including cytochromes P450, which metabolize drugs [67,68,69].

## 3. Materials and Methods

### 3.1. Reagents

Glycol chitosan 72 kDa (GlycChit, the degree of deacetylation is 92), mannan (46 kDa), polyethyleneimine 1.8 kDa (PEI1.8), Et_2_O, DMF, DMSO, HBr, (HOCH2CH2)3N, 2-hydroxypropyl-β-cyclodextrin (HPCD), 4-toluenesulfonyl chloride (TsCl), genipin, 1-Ethyl-3-(3-dimethylaminopropyl) carbodiimide (EDC), N-hydroxysuccinimide (NHS), N-acetylcysteine, limonene were purchased from Sigma Aldrich (St. Louis, MI, USA). Mannotriose-di-(N-acetyl-D-glucosamine) (triMan-GlcNAc2) was obtained from Dayang Chem (Hangzhou, China) Co., Ltd.

Using CD spectroscopy (Jasco J-815 CD Spectrometer, JASCO Corp., Tokyo, Japan), the degree of deacylation in glycol chitosan samples was determined by the peak at 215 nm corresponding to the absorption of the amide bond and was 92%.

Moxifloxacin hydrochloride (MF) was obtained by Canon Pharma (Moscow, Russia).

Linalool was purchased from Carl Roth GmbH (Karlsruhe, Germany). Eugenol at the highest commercial quality was purchased from Acros Organics (Geel, Belgium). Apiol with 98–99% purity was obtained by high-efficiency distillation using a pilot plant device at N.D. Zelinsky Institute of Organic Chemistry RAS (Moscow, Russia) [42].

The components of the LB medium were bactotrypton, agarose and yeast extract (Helicon, Russia).

### 3.2. HPCD–PEI1.8–triMan Polymer Synthesis and Properties

Activated HPCD was obtained as described earlier [6,65] by the reaction of HPCD OH groups with carbonyldiimidazole in DMSO. We mixed 100 mg of PEI1.8 and 200 mg of triMan-GlcNAc2, followed by dissolution in 10 mL of 10 mM HCl. NaOH was added to the sample to achieve pH 6–7, and NaBH3CN to reduce the Schiff base. The mixture was incubated at 70 °C for 48 h. Then, a 5-fold molar (on PEI1.8) excess of activated HPCD was added to the mixture, followed by incubation at 70 °C for 24 h. Polymer purification was carried out by dialysis against water for 24 h with water replacement (cut-off 3.5 kDa) and centrifugation (cut-off 3 kDa). The polymer was freeze-dried at –60 °C (Edwards 5, BOC Edwards, Burgess Hill, UK). The degree of purity of the conjugates was controlled by reverse-phase HPLC with elution with 1 mL/min of acetonitrile–water (80/20 *v*/*v*). The chromatograms are presented in the Supplementary Material (Figure S2). The degree of mannosylation and HPCD grafting was calculated according to spectrophotometric titration of amino groups (before and after reaction) with 2,4,6-trinitrobenzenesulfonic acid [6,17] and with FTIR and NMR data. Mainly, primary amino groups were detected.

Polymer properties: HPCD-PEI1.8–triMan is characterize by a molecular weight of 10 ± 3 kDa, with a corresponding molar ratio of the components of 3:1:5, a hydrodynamic size of 120 ± 30 nm, a zeta potential of +5 ± 1 mV.

### 3.3. MF Conjugation with Polymers

We used HPCD–PEI1.8–triMan, glycol chitosan (GlycChit), and mannan to obtain covalent conjugates with MF (MF1–MF3).

**MF1**. The synthesis was carried out based on the reaction (Figure 1) described by authors [70]. In this reaction, 130 mg of MF was mixed with EDC (150 mg) and NHS (65 mg), dissolved in 15 mL of PBS (0.01 M, pH 7.4). The mixture was incubated for 30 min at 70 °C. Then, 45 mg of HPCD–PEI1.8–triMan was added and stirred for 20 h at 70 °C. Conjugate purification was carried out by dialysis against water for 24 h with water replacement (cut-off 6–8 kDa) and centrifugation (cut-off 10 kDa).

**MF2** and **MF3**. The synthesis was carried out based on the reaction (Figure 1b) described in other works, with modifications [71,72]. MF (100 mg) was mixed with TsCl (1.1 molar excess) and dissolved in 2 mL of DMF. The mixture was incubated for 1 h at 80 °C. Then, glycol chitosan (GlycChit) or mannan (25 mg) was added and incubated for another 24 h at 80 °C. After that, 1 mL of Et_2_O was added to the cooled solutions to precipitate the product. Et_2_O was evaporated, followed by dissolution in water and centrifugation (cut-off 10 kDa).

The content of MF in all samples was determined by reading the A290. The samples were frozen and freeze-dried as described in Section 3.2.

### 3.4. Preparation of MF Non-Concovalent Formulations 

**MF-gel1**. We mixed 20 mg of MF with 7 mg of GlycChit, and 0.5 mg of genipin was added. The mixture was dissolved in 3 mL of PBS and incubated for 24 h at 60 °C. Genipin stitched the chitosan chains, as shown in previous works [45,46,47,48,49,50,73] and loaded the MF molecules.

**MF-gel2**. We reacted 40 mg of GlycChit with 110 mg of N-acetylcysteine, similarly to what described for MF1. GlycChit-ACC was mixed with MF in a mass ratio of 1:3 with subsequent incubation at 50 °C in PBS for 2 h after the addition of 5 mg of oxidized glutathione for S-S crosslinking of the GlycChit-ACC chains.

Using an atomic force microscope (AFM microscope INTEGRA II), the particle size of the nanogels obtained by crosslinking with genipine and acetylcysteine were found to be 200–350 nm.

### 3.5. Adjuvant Conjugation with Polymers

We added 0.5 mL of HBr (48%) to 0.1 g of eugenol (EG), apiol, linalool or limonene [15]. The mixture was incubated for a day at 50 °C. Then, 0.5 mL of Et_2_O and 0.5 mL of H_2_O were added to the cooled solution for extraction. The ether from the extract was evaporated, and then HPCD–PEI1.8–triMan (polymer/adjuvant = 1:5 *w*/*w*) was added to the Br-modified substances in PBS. The samples were incubated at 70 °C for 12 h. Conjugate purification was carried out by centrifugation (cut-off 10 kDa).

### 3.6. FTIR Spectroscopy

The FTIR spectra of samples were recorded using a Bruker Tensor27 spectrometer equipped with a liquid nitrogen-cooled MCT (mercury cadmium telluride) detector, as described earlier [6,32,74,75].

### 3.7. NMR Spectroscopy

We dissolved 10–15 mg of the samples in 700 μL of D_2_O. The ^1^H and ^13^C NMR spectra of the solutions were recorded on a Bruker Avance 400 spectrometer (Bruker Biospin, Rheinstetten, Germany) at an operating frequency of 400 MHz. The chemical shifts are shown in ppm on the δ scale relative to hexamethyldisiloxane as an internal standard. The analysis and processing of the NMR spectra were performed with the program MestReNova v.12.0.0–20080).

### 3.8. Antibacterial Activity Studies: FTIR Spectroscopy, Microbiology

The strains used in this study were *Escherichia coli* (ATCC 25922) and *Bacillus subtilis* (ATCC 6633) from the National Resource Center Russian collection of industrial microorganisms, SIC “Kurchatov Institute”. The cultures were cultivated for 18–20 h at 37 °C to a CFU/mL ≈ 10^7^ (determined by measuring A_600_) in the liquid nutrient medium Luria–Bertani (pH 7.2): *E. coli*—without stirring, *B. subtilis*—with stirring 100 rpm.

The ATR-FTIR spectra of cell suspension samples were recorded using the following procedure: overnight cell suspensions (10^7^ CFU/mL) were washed twice with sterile PBS (pH 7.4) to eliminate the culture medium by centrifuging (Eppendorf centrifuge 5415C, 5 min, 10,000× *g*). The cells were precipitated by centrifugation and separated from the supernatant, washed twice and resuspended in PBS (10^8^ cells/mL) to record the FTIR spectra. The cell suspensions were incubated with the MF-containing samples, and the FTIR spectra were recorded at 37 °C online.

Microbiologic studies. The cultures were cultivated for 18–20 h at 37 °C to a CFU ≈ 0.3 × 10^8^ (colony-forming unit) in liquid nutrient medium Luria–Bertani (pH 7.2). The experiments in liquid medium were conducted by adding 50 μL of the samples to 5000 μL of cell culture. The specimens were incubated at 37 °C for seven days. At the specific time, 300 μL of each sample was taken and diluted with PBS, and the absorbance was measured at 600 nm (with CFU control). For a quantitative analysis of the dependence of CFU (cell viability) on the concentration of MF, 50 μL of each sample was diluted 10^6^–10^8^ times and seeded on Petri dishes. The dishes were placed in the incubator at 37 °C for 24 h. Then, the number of colonies (CFU) was counted. The number of living cells was additionally determined by measuring the fluorescence of the DAPI dye, relative to obviously dead cells and living ones, after 10 min of incubation of 200 µL of a cell suspension sample with 1 µg/mL of DAPI.

## 4. Conclusions

An urgent issue of modern medicine is the creation of combined drug formulations based on natural safe substances that replace toxic antibiotics. Natural extracts from essential oils such as allylbenzenes and terpenoids are important biologically active substances that are promising for the creation of new drug formulations with attractive functionalities, including the inhibition of drug efflux in bacterial or eukaryotic cells, as well as the inhibition of enzymes (such as cytochrome P450) that metabolize the main active substances. Indeed, recently, several reports demonstrated that the concomitant administration of natural extracts from essential oils may affect drug metabolism in humans, enhancing the bioavailability and prolonging the metabolic elimination of many drugs, thereby significantly increasing their plasma concentration. In this work, EG, apiol, linalool and limonene conjugated with polymers (so that their use would be possible in aqueous solutions) were studied as enhancers of the action of the fluoroquinolone MF against *E. coli* and *B. subtilis*. We obtained MF-prodrug and adjuvants both covalently attached to polymers (dendrimers) based on PEI, cyclodextrins, chitosan or mannan. As a control, drug compositions included in nanogels through the crosslinking of the polymer chains were created, so as not to modify the drug functional groups. The polymer–MF demonstrated an increase in the antibacterial effect of MF, with a decrease in the minimum inhibitory concentration by 2–5 times, an increase in the penetration of MF into the cells, as well as a prolonged duration up to 10 days (versus 2–3 days for simple MF).

We presented an original technique for tracking the interaction of a drug and a delivery system with bacterial cells using FTIR spectroscopy, which provides valuable information about the structural components of a cell. So, we showed that adjuvant–polymer (covalent) formulations increased the MF influx due to membrane permeability enhancement and efflux inhibition. The covalent conjugates, as well as polymer particles with the addition of apiol (cytochrome P450 inhibitor) improved the pharmacokinetic parameters of MF: the half-life increased by 2–5 times, while the area under the kinetic curve (AUC), which characterizes the effective concentration of a drug in the blood plasma, increased up to 8–10 times. Thus, the covalent formulations of MF and adjuvants (terpenoids and allylbenzenes) have the potential to overcome multidrug resistance and promote the long-term action of MF, as a result of increased penetration of MF into bacterial cells due to the adjuvant and polymer, increased accumulation of the drug in the pathogen localization sites due to the targeting of the macrophage mannose receptors and increased half-life of the drug due to the polymer shell and the blocking of metabolizing enzymes.

## Figures and Tables

**Table 1:** Chemical composition and MF content in the conjugates.

Designation	Chemical Composition	MF Percentage *, %
MF	MF free	100
MF1	MF–HPCD–PEI1.8-triMan (covalent), 13 MF molecule on polymer	34 ± 5
MF2	MF–GlycChit (covalent), 1 MF molecule accounts for 2.4 glucosamine unit	45 ± 3
MF3	MF–Mannan (covalent), 1 MF molecule accounts for 5.3 mannose unit	32 ± 2
MF-gel1	MF + GlycChit genipin-stitched (non-covalent)	72 ± 4
MF-gel2	MF + GlycChit-acetylcysteine S-S stitched (non-covalent)	69 ± 3

* MF percentage was determined by A290.

**Table 2:** The concentration of MF-containing drugs required for 50% inhibition of cell growth (IC50).

Designation *	IC50 against *E. coli*, ng/mL	IC50 against *B. subtilis*, ng/mL
MF	8 ± 2	800 ± 300
MF1	40 ± 7	11 ± 3
MF2	4 ± 1	9 ± 2
MF3	3 ± 1	10 ± 3
MF-gel1	7 ± 2	20 ± 5
MF-gel2	7 ± 2	15 ± 4

* The chemical designations of MF conjugates, adjuvants and polymers are indicated in Table 1.

**Table 3:** Cell viability (as a percentage of CFU with respect to the control, determined by DAPI cell staining) for formulations based on allylbenzenes, terpenes and terpenoids. The concentration of EG, apiol, linalool, limonene was 1 mg/mL.

	*E. coli*			*B. subtilis*		
Compound X	X-Covalent with HPCD–PEI1.8–triMan (X-Polymer)	MF Free 30 ng/mL * + X-Covalent with HPCD–PEI1.8–triMan	X + GlycChit Genipin-Stitched (X-gel)	X-Covalent with HPCD–PEI1.8–triMan (X-Polymer)	MF Free 30 ng/mL ** + X-Covalent with HPCD–PEI1.8–triMan	X + GlycChit Genipin-Stitched (X-Gel)
EG	64 ± 7	41 ± 3	85 ± 11	81 ± 8	60 ± 7	94 ± 3
Apiol	75 ± 9	44 ± 5	92 ± 8	81 ± 7	59 ± 7	93 ± 4
Linalool	85 ± 6	47 ± 4	93 ± 7	85 ± 9	62 ± 5	97 ± 2
Limonene	74 ± 9	46 ± 2	94 ± 5	83 ± 6	59 ± 7	89 ± 5

* For free MF at 30 ng/mL, 57% cell viability was observed. ** For free MF at 30 ng/mL, 75% cell viability was observed.

**Table 4:** Pharmacokinetic parameters of MF determined for white mongrel rats. Drugged animals. The study time was 6 h. Two-compartment model without absorption.

Parameters	MF Free	MF + HPCD–PEI1.8–triMan (Non-Covalent)	MF + EG + HPCD–PEI1.8–triMan (Non-Covalent)	MF + Apiol + HPCD–PEI1.8–triMan (Non-Covalent)	MF–Mannan (Covalent)	MF + Mannan (Mixture)
Half-distribution period, min	20 ± 2	15 ± 1	12 ± 2	25 ± 4	15 ± 2	14 ± 2
Half-elimination period, h	6.5 ± 0.5	10 ± 1	22 ± 2	45 ± 6	42 ± 7	24 ± 3
Kinetical distribution volume, L	8 ± 1	4 ± 1	4 ± 1	3 ± 1	3 ± 1	3 ± 1
Stationary distribution volume, L	4 ± 1	4 ± 1	4 ± 1	3 ± 1	3 ± 1	3 ± 1
Clearance, mL/min	14 ± 2	4.5 ± 0.6	2.0 ± 0.4	0.9 ± 0.2	0.8 ± 0.1	1.3 ± 0.3
Area under curve	360 ± 70	1100 ± 200	2400 ± 300	5800 ± 700	6500 ± 900	3800 ± 500
Mean residence time, h	5 ± 1	14 ± 2	28 ± 4	35 ± 6	35 ± 4	29 ± 3

## Data Availability

Data are contained within the article and the Supplementary Materials.

## Supplementary Materials

The following supporting information can be downloaded at: https://www.mdpi.com/article/10.3390/ph16081102/s1, Figure S1. (a) ^13^C NMR spectra of MF–HPCD–PEI1.8–triMan. (b) ^1^H NMR spectra of Linalool-Br (product of HBr addition to linalool). (c) ^13^C NMR spectra of Linalool-Br (product of HBr addition to linalool). (d) ^1^H NMR spectra of EG-Br (product of HBr addition to EG). (e) ^13^C NMR spectra of EG-Br (product of HBr addition to EG). D_2_O. T = 25 °C. Figure S2. HPLC diagrams of MF conjugates with polymers. Knauer chromatography system (Knauer, Berlin, Germany) on a Diasfer-110-C18 column (BioChemMack, Moscow, Russia): grains—6 μm, size 4 × 150 mm. The eluent was CH_3_CN-H_2_O (80:20, *v*:*v*); the elution rate was 1 mL/min, 22 °C. Detection was performed by MF absorption at 290 nm.

## Abbreviations

EG	eugenol
GlycChit	glycol chitosan
HPCD	(2-hydroxypropyl)-β-cyclodextrin
IC50	Half-maximal inhibitory concentration
MF	moxifloxacin
PEI	polyethyleneimine
triMan	mannotriose residue
