# Supplementary material for: Covalent Conjugates of Allylbenzenes and Terpenoids as Antibiotics Enhancers with the Function of Prolonged Action
Source: Pharmaceuticals (Basel). 2023 Aug 4;16(8):1102. doi: 10.3390/ph16081102 (PMC10459265; doi:10.3390/ph16081102)

Content

Figure S1. (a) ^{13}C NMR spectra of MF-HPCD-PEI1.8-triMan. (b) ^{1}H NMR spectra of Linalool-Br (product of HBr addition to linalool). (c) ^{13}C NMR spectra of Linalool-Br (product of HBr addition to linalool). (d) ^{1}H NMR spectra of EG-Br (product of HBr addition to EG). (e) ^{13}C NMR spectra of EG-Br (product of HBr addition to EG). D_2O. T = 25 °C.

Figure S2. HPLC diagrams for MF conjugates with polymers. Knauer chromatography system (Knauer, Berlin, Germany) on Diasfer-110-C18 column (BioChemMack, Moscow, Russia): grains – 6 μm, size 4×150 mm. The eluent was CH_3CN-H_2O (80:20, v:v); the elution rate was 1 mL/min, 22 °C. Detection was performed by MF absorption at 290 nm.

Figure S1. (a) ^{13}C NMR spectra of MF-HPCD-PEI1.8-triMan. (b) ^{1}H NMR spectra of Linalool-Br (product of HBr addition to linalool). (c) ^{13}C NMR spectra of Linalool-Br (product of HBr addition to linalool). (d) ^{1}H NMR spectra of EG-Br (product of HBr addition to EG). (e) ^{13}C NMR spectra of EG-Br (product of HBr addition to EG). D_2O. T = 25 °C.

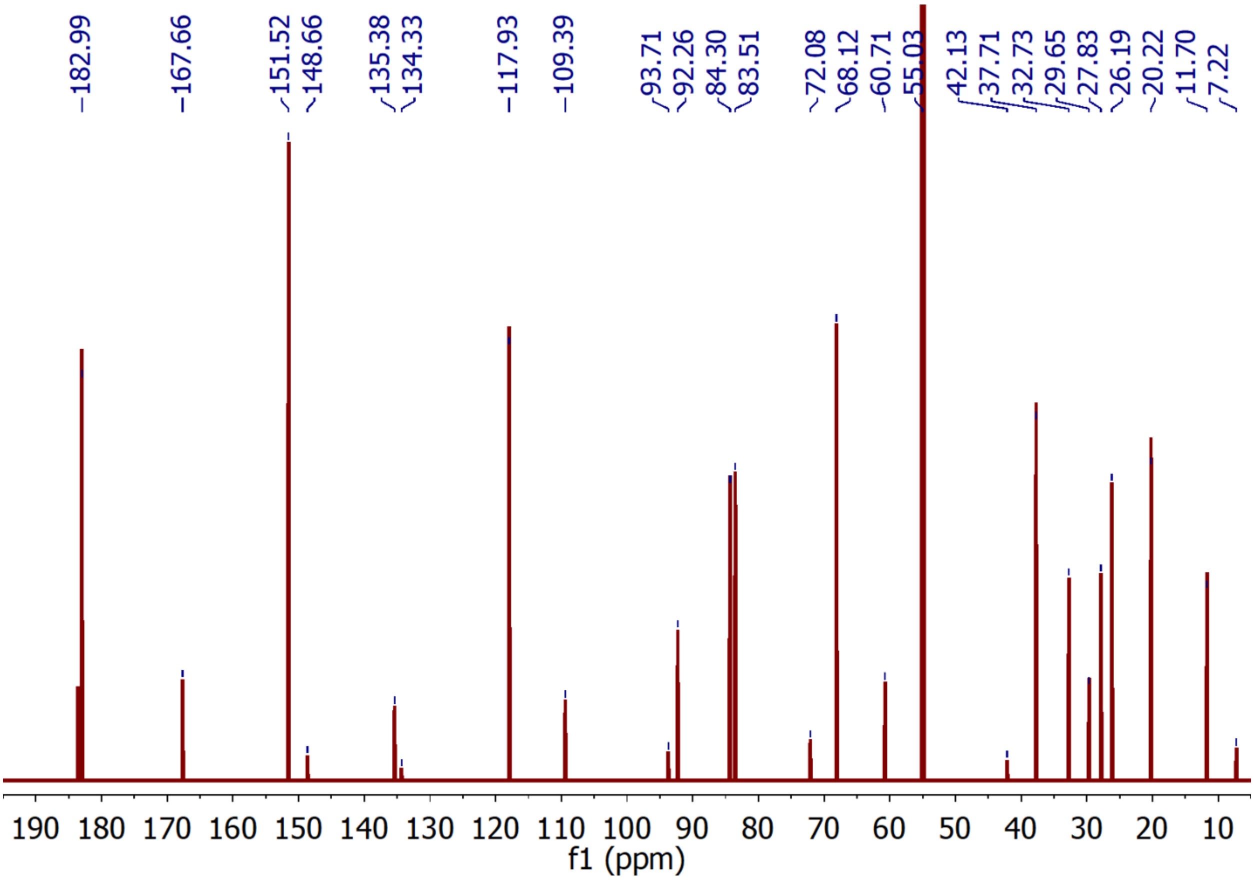

(a)

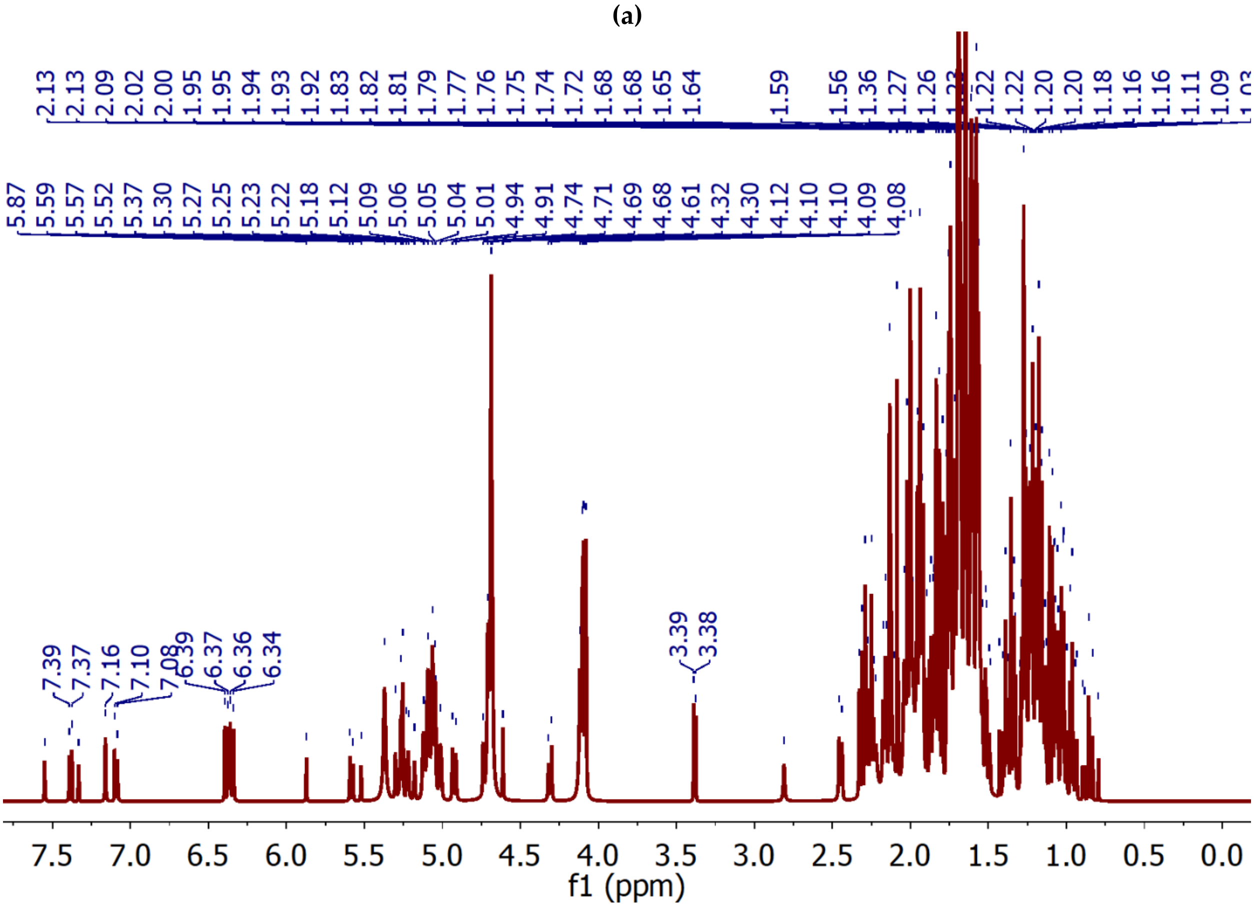

(b)

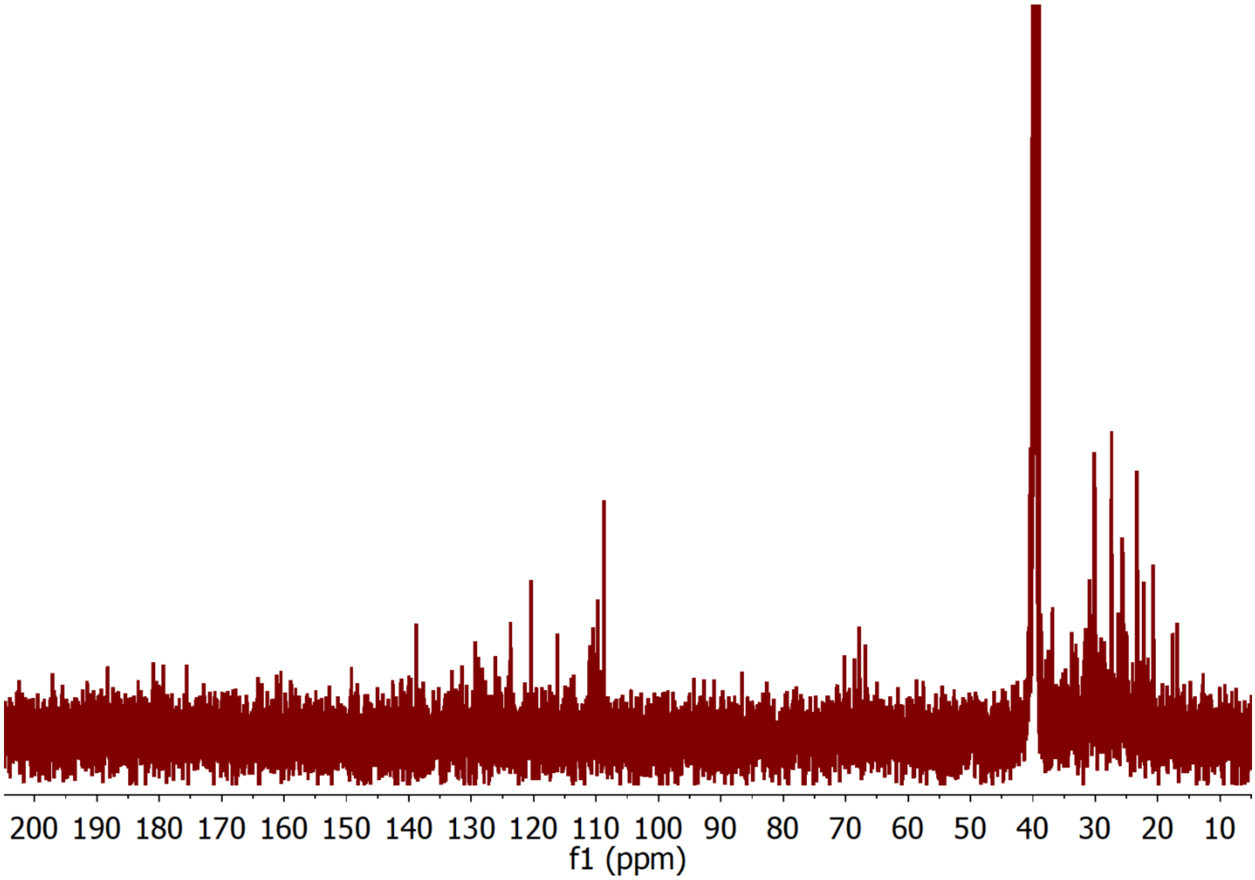
200 190 180 170 160 150 140 130 120 110 100 90 80 70 60 50 40 30 20 10
f1 (ppm)
(c)

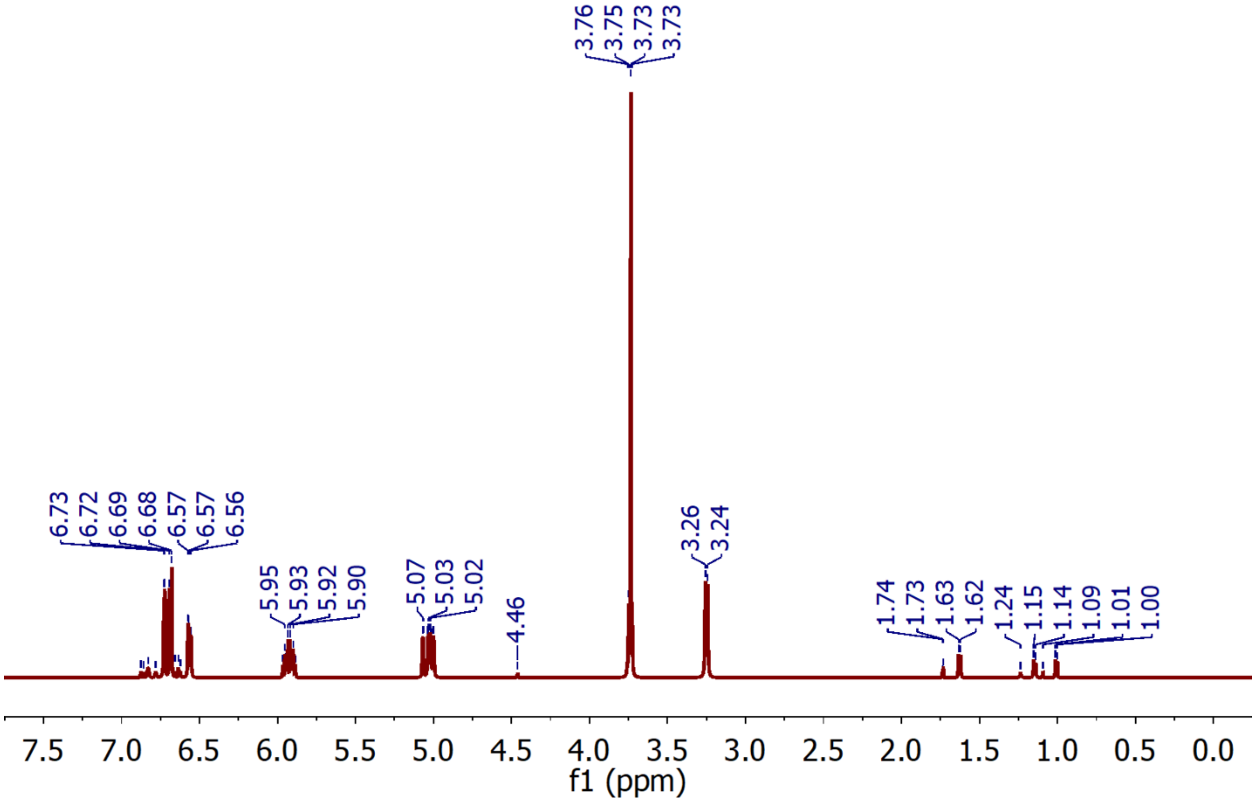
3.76
3.75
3.73
3.73
6.73
6.72
6.69
6.68
6.57
6.57
6.56
3.26
3.24
5.95
5.93
5.92
5.90
5.07
5.03
5.02
4.46
1.74
1.73
1.63
1.62
1.24
1.15
1.14
1.09
1.01
1.00
7.5 7.0 6.5 6.0 5.5 5.0 4.5 4.0 3.5 3.0 2.5 2.0 1.5 1.0 0.5 0.0
f1 (ppm)
(d)

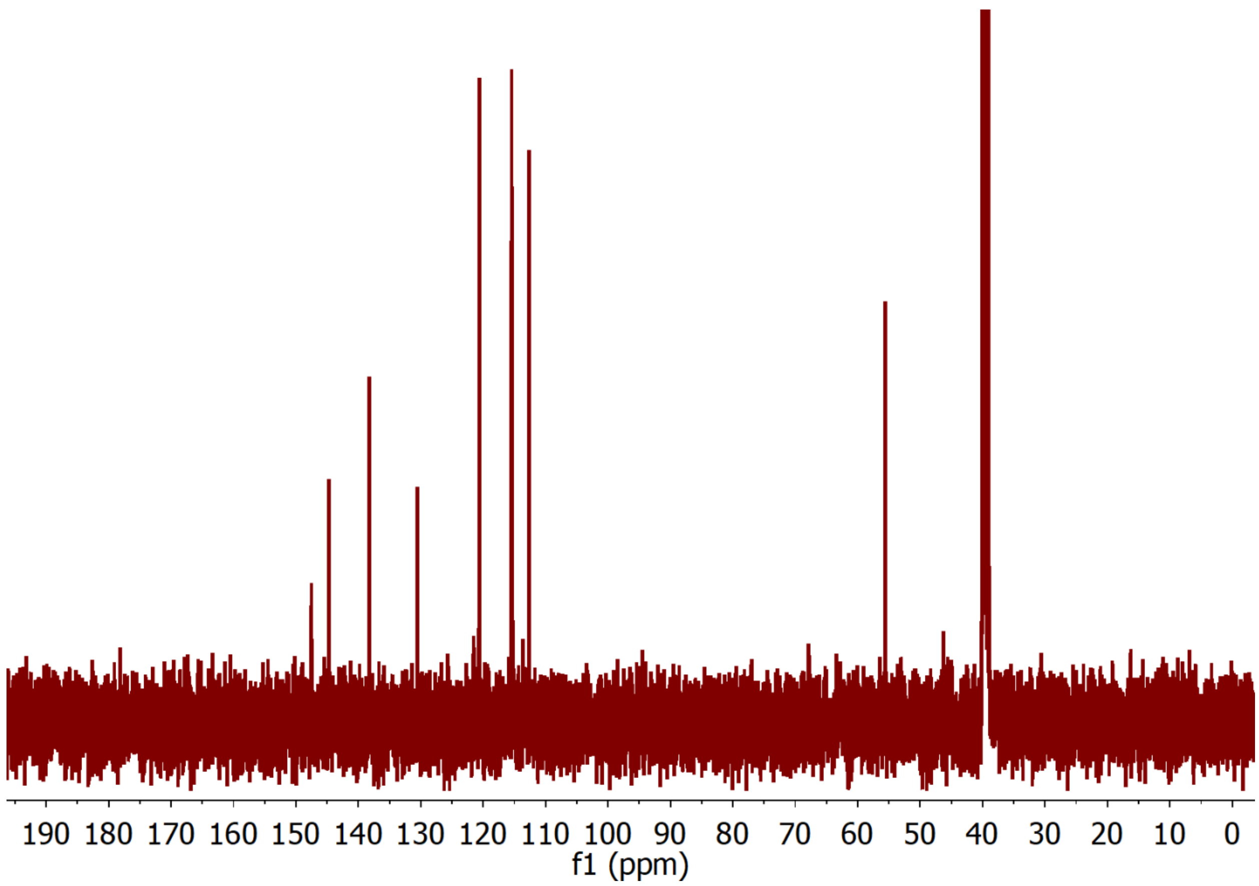
190 180 170 160 150 140 130 120 110 100 90 80 70 60 50 40 30 20 10 0
f1 (ppm)

(e)

Figure S2. HPLC diagrams for MF conjugates with polymers. Knauer chromatography system (Knauer, Berlin, Germany) on Diasfer-110-C18 column (BioChemMack, Moscow, Russia): grains – 6 μm, size 4×150 mm. The eluent was CH_3CN-H_2O (80:20, v:v); the elution rate was 1 mL/min, 22 °C. Detection was performed by MF absorption at 290 nm.

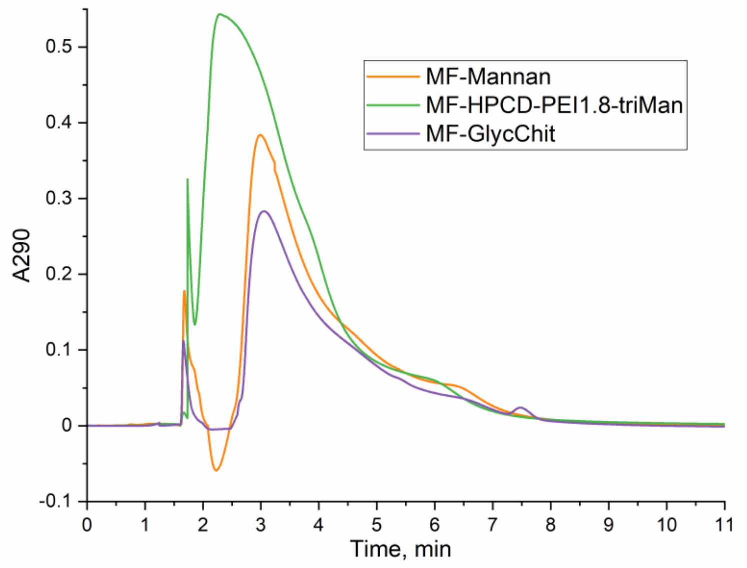

Supplement: Supplementary file 1 [file pharmaceuticals-16-01102-s001.zip › pharmaceuticals-2510116-supplementary.pdf]
